# Effects of weight loss on bone turnover, inflammatory cytokines, and adipokines in Chinese overweight and obese adults

**DOI:** 10.1007/s40618-022-01815-5

**Published:** 2022-05-30

**Authors:** D. Yu, W. Chen, J. Zhang, L. Wei, J. Qin, M. Lei, H. Tang, Y. Wang, S. Xue, J. Dong, Y. Chen, L. Xie, H. Di

**Affiliations:** 1 Department of Nutrition, The Third Hospital of Hebei Medical University, Shijiazhuang, China; 2 Department of Orthopedic Surgery, The Third Hospital of Hebei Medical University, Shijiazhuang, China; 3 Clinical Biochemistry Lab, The Third Hospital of Hebei Medical University, Shijiazhuang, China; 4 Department of Nuclear Medicine, The Third Hospital of Hebei Medical University, Shijiazhuang, China; 5 The Biobank, The Third Hospital of Hebei Medical University, Shijiazhuang, China; 6 Joint Department, The Third Hospital of Hebei Medical University, Shijiazhuang, China; 7 The Graduate School, Hebei Medical University, Shijiazhuang, China; 8 School of Chemical Engineering, Shijiazhuang University, Shijiazhuang, China

**Keywords:** Weight loss, Obesity, Bone turnover, Inflammatory cytokines, Adipokines

## Abstract

**Purpose:**

Plenty of studies have examined the long term effect of weight loss on bone mineral density. This study aimed to explore the effects of 10% weight loss on early changes in bone metabolism as well as the possible influencing factors.

**Methods:**

Overweight and obese outpatients (BMI > 24.0 kg/m^2^) were recruited from the nutrition clinic and followed a calorie-restricted, high-protein, low-carbohydrate diet program. Dietary intake, body composition, serum procollagen type I N-propeptide (PINP), β-Crosslaps, PTH, 25(OH) VitD, a series of inflammatory cytokines and adipokines were measured for the participants before starting to lose weight and after 10% weight loss (NCT 04207879).

**Results:**

A total of 75 participants were enrolled and 37 participants achieved a weight loss of at least 10%. It was found that PINP decreased (*p* = 0.000) and the β-Crosslaps increased (*p* = 0.035) in female participants. Decreases in PTH (*p* = 0.001), serum IL-2 (*p* = 0.013), leptin (*p* = 0.001) and increases in 25(OH) VitD (*p* = 0.001), serum ghrelin (*p* = 0.033) were found in 37 participants after 10% of their weight had been lost. Change in PINP was detected to be significantly associated with change in lean body mass (*r* = 0.418, *p* = 0.012) and change in serum ghrelin(*r* = − 0.374, *p* = 0.023).

**Conclusions:**

Bone formation was suppressed and bone absorption was increased in female subjects after a 10% weight loss. Bone turnover was found to be associated with lean body mass and affected by the circulating ghrelin level.

**Supplementary Information:**

The online version contains supplementary material available at 10.1007/s40618-022-01815-5.

## Introduction

Weight reduction is recommended to alleviate the comorbidities linked to conditions of overweight and obesity. As little as a 5% reduction in body weight has been documented to diminish the risk of all-cause mortality [1, 2]. Loss in weight is recognized to result in a reduction in lean body mass; thus, growing research evidences have explored changes in bone mass as a response to loss in weight among overweight along with obese individuals. In these investigations, most researchers have focused on the long-term influences of weight loss on bone mineral density (BMD), which is widely used for the assessment of osteoporosis. However, because BMD is a static parameter, it requires a minimum of six months before observing remarkable changes. Actually, in adults, bone tissue goes through constant process of remodeling every second; this is indicated by the detection of bone turnover biomarkers after 2–3 months of weight loss treatment [3, 4]. Thus, in this study, we employed bone turnover markers to examine the rapid changes which might occur in early stages of weight loss treatment.

Remodeling, or bone turnover, takes place via a coupled process of bone re-sorption and bone formation. In recent years, several global osteoporosis guidelines have recommended the use of serum procollagen type I N-propeptide (PINP), as well as β-Crosslaps (β-CTX) as blood reference markers for bone formation and bone re-sorption, respectively [5–8]. In addition, bone-metabolism-linked biochemical biomarkers consisting of calcium along with phosphorus in serum, as well as urine, bone metabolism modulatory hormones, for instance PTH (parathyroid hormone), vitamin D along with their metabolites have been recognized as good indices for rapid identification of the status of human bone loss [9].

The relationship of weight loss with bone mass change is intricate and complex, involving an integration of mechanical, endocrine, and cytokine modulations [10]. In general, after a calorie-restricted diet treatment, the serum concentrations of inflammatory cytokines, for instance IL-2, TNF-α, IL-6, IL-10, adipocyte-derived hormones, leptin, adiponectin, and ghrelin change in line with the decreases in total weight and adiposity. However, scant data are currently available on how these inflammatory cytokines and adipokines correlate with bone formation along with bone resorption in obese subjects in the course of weight loss. In addition, no previous research has examined the correlation between weight loss rates and changes in bone turnover. To address these knowledge gaps, we performed a clinical investigation to explore the effects of a 10% weight loss on bone metabolism and the relationships of changes in bone turnover with the changes in inflammatory cytokines and adipokines among overweight, as well as obese adults.

## Methods

This study was undertaken at the Department of Nutrition in the Third Hospital of Hebei Medical University and was registered on ClinicalTrials.gov (NCT04207879).

### Participants

Overweight and obese adults (BMI > 24.0 kg/m^2^) who visited the nutrition clinic between 1 March 2019 and 31 August 2021 were screened for inclusion. Inclusion criteria included: (1) 18–55 years of age; and (2) a stable body weight (weight loss or weight gain < 3 kg) for at least 3 months prior to enrollment. Exclusion criteria included: (1) the concomitant utilization of any medication for weight loss (e.g., Orlistat) or regime of diet/exercise designed for loss of weight; (2) previous bariatric or other intestinal surgery; (3) pregnancy or lactation; (4) clinically remarkable cardiovascular or respiratory diseases; (5) impaired renal function (defined as an estimated glomerular filtration rate < 90 ml min^–1^ per 1.73 m^2^) and impaired liver function(defined as liver enzymes greater than equal to twofold the upper normal limit); (6) active malignancy; (7) chronic infections; (8) hypothyroidism; (9) Cushing’s disease; (10) the use of lipid-lowering or diabetes medication; (11) chronic utilization of antacids; (12) the use of antibiotics within the three months prior to enrollment; (13) alcohol or drug addiction; (14) change in smoking habits within the previous 3 months or a plan to quit smoking in the following days.

### Study design

After finishing screening and including the participants, we collected baseline information, including inquiries about medical history, a physical examination, and co-morbidity evaluation for each included subject; then, dietary intake assessments, anthropometric measurements, body composition analyses, and fasting blood samplings were performed at baseline.

Then, all subjects entered a program that was medically supervised to reduce body weight by 10% or more. Weight loss was achieved through the prescription of a hypocaloric, high-protein, low-carbohydrate diet. At the baseline, most participants were sedentary and were asked to continue their usual physical activity levels throughout the study.

Thereafter, we closely kept track of the weight of each subject. All subjects were asked to self-report their fasting weight via a mobile phone application in the morning. Upon reaching a 5% loss of the initial body weight, subjects were asked to re-visit the outpatient clinic and underwent a face-to-face investigation, which included a detailed dietary survey, a GPAQ physical activity questionnaire, anthropometric measurements, and body composition analysis. When subjects reached a 10% weight loss, they were required to come back again and complete an identical information collection procedure; in addition, each subject’s fasting venous blood was collected for laboratory analysis.

### Weight loss treatment

Upon entry to the study, all subjects were required to follow a uniform diet plan, which was a hypocaloric diet with a structured advice for daily macronutrient consumption (total energy was equal to basal metabolism, consisting of 35% total energy (E) intake from carbohydrate, 25% E from protein, and 40% E from fat). For each participant, an individualized, detailed 7-day recipe, including all recommended and unrecommended foods and beverages, was formulated by the registered dietitian. Participants were instructed to consume only the recommended foods or beverages during the following days. All participants were required to send pictures of their meals to the dietitian via WeChat every day, and the dietitian gave timely comments and recommendations to make sure the participants complied to the required diet pattern. At the same time, any adverse events were reported immediately via the app or phone call.

### Dietary intake assessment

By utilizing the information of a 24 h recall diet survey, which was performed by trained dietitians face to face, daily nutrient intakes were determined by using the individualized evaluation software for the nutrition clinic (version 1.0, Shanghai Zhending Health Technology Co., Ltd). The individual daily intakes of macronutrients (carbohydrate, fat, and protein) and total energy were then collated for assessment of compliance to the planned diet.

### Anthropometric measurement along with body composition analyses

Body weight along with body composition was measured following a 12 h fast in light clothing at each study visit. The measurements were conducted by BIA (bioimpedance analysis) (InBody S10; Biospace). All BIA assessments were carried out by the same trained laboratory technician. We determined the height at baseline via a wall-mounted audiometer. BMI was computed via dividing the weight (kg) with the height squared (m^2^).

### Laboratory assay

#### Bone metabolism indicators

Fasting serum concentrations of β-Crosslaps (Elecsys β-Crosslaps/serum) and total P1NP (Elecsys total P1NP) were measured with a Modular Biochemical Immunoassay System (Cobas e601, Roche Diagnostics Pty (Ltd.), Johannesburg, South Africa). Serum 25(OH) VitD and parathyroid hormone (PTH) were assessed via chemiluminescent immunoassay (MAGLUMI 4000 Plus, Xinchanye Biomedical Engineering Co., Ltd., Shenzhen, China). The intra-assay CV (coefficients of variation) were 3.5% for β-Crosslaps, 2.6% for total P1NP, < 10% for 25(OH) VitD, and < 5% for PTH, and the inter-assay CVs were 8.4%, 4.1%, < 15%, and < 10%, respectively.

#### Inflammatory cytokines

High-sensitivity IL-1β, IL2, IL6, IL8, IL10, and TNF-α were assessed via absorbance enzyme-linked immunosorbent assay (ELISA) methods (ExCell Bio, Shanghai, China). The manufacturer-reported sensitivities (via the average minimum detection dosage), standard level ranges, and inter- and intra-assay CVs for each biologic signature: IL-1β: 4.0 pg/mL, 0–500 pg/mL, < 10%, < 10%; IL-2: 7.0 pg/mL, 0–500 pg/mL, < 10%, < 10%; IL-6: 1.0 pg/mL, 0–250 pg/mL, < 10%, < 10%; IL-8: 2.0 pg/mL, 0–500 pg/mL, < 10%, < 10%; IL-10: 7.0 pg/mL, 0–1000 pg/mL, < 10%, < 10%; TNF-α: 7.0 pg/mL, 0–1000 pg/mL, < 10%, < 10%.

#### Adipokines

Leptin along with adiponectin were measured using absorbance ELISA methods (ExCell Bio, China, CN). The manufacturer reports sensitivities for leptin and adiponectin detection of 30 pg/mL and 100 pg/mL, respectively; the standard concentration ranges for leptin and adiponectin detection are 0–4000 pg/mL and 0–20,000 pg/mL, respectively. The inter- along with intra-assay CVs for the these biomarkers are both below 10%. Ghrelin (acylated) was measured using absorbance enzyme-linked immunosorbent assay (ELISA) methods (Quantikine kits; RayBiotech, Morcross, GA, USA). The manufacturer-reported sensitivity for detection is 8 pg/mL, and the standard concentration range is 10–10,000 pg/mL. The intra-assay CV% is below 10%, and the inter-assay CV% is below 12%.

### Statistical analysis

Data were tested for normal distribution with the Shapiro–Wilk test and QQ plots. Descriptive statistics are given as the mean and SD (data normally distributed) or the median and interquartile range (data non-normally distributed). Paired *t* tests or Wilcoxon’s tests were conducted to examine the differences in repeated measured dietary intakes, body composition parameters, bone metabolism indicators, inflammatory cytokines, and adipokines. Spearman correlation coefficients were calculated to evaluate the relationships between changes in bone metabolism indicators and changes in fat body mass, lean body mass, calcium intake, weight loss velocity, inflammatory cytokines, and adipokines from baseline to 10% weight loss. Changes in the above parameters were expressed as Δ variables, computed via subtracting the values measured after 10% weight was lost from the values at baseline. Δchange% was computed via dividing the value of Δchange by the value at baseline. Statistical analyses were implemented in the SPSS v26 software program. *p* < 0.05 signified statistical significance.

## Results

**Fig. 1:**
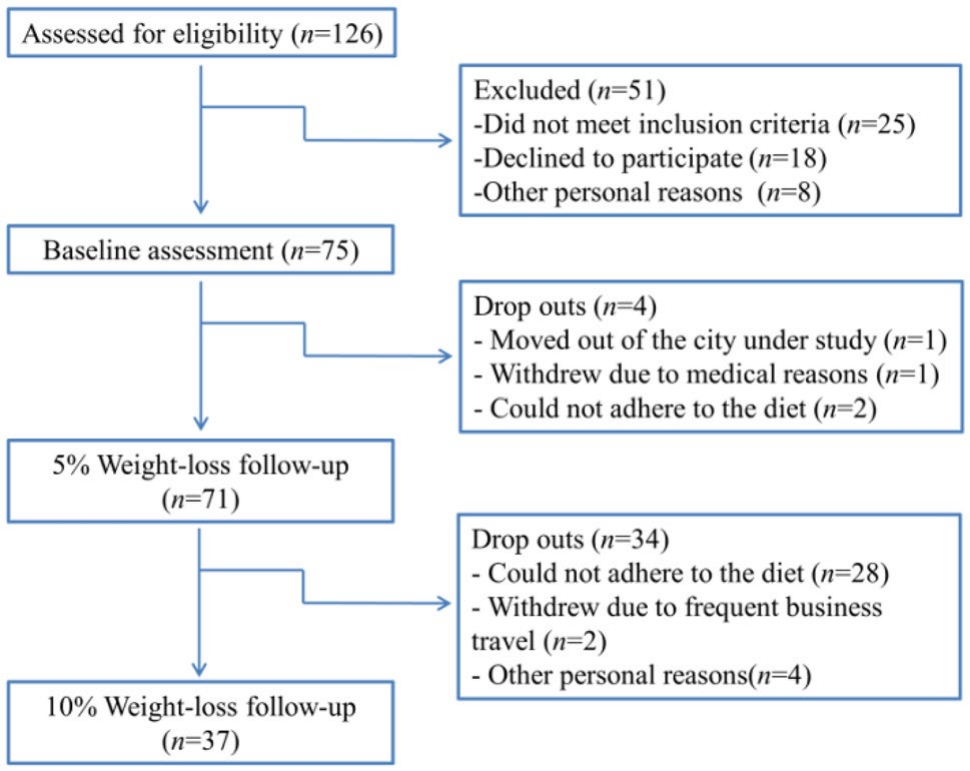
Flowchart for the study

A total of 126 overweight and obese outpatients were assessed for eligibility to participate in this study; among them, 75 subjects were enrolled who satisfied the inclusion and none of the exclusion criteria. Of the 75 subjects, 4 dropped out before achieving a 5% reduction in body weight, with the remaining 71 participants attaining a weight loss of at least 5%, and eventually, 37 participants attaining a weight loss of at least 10% (see Fig. 1). Thus, the 37 subjects who successfully achieved a 10% weight loss were included for final data analyses.

Of the final 37 subjects (male, *n* = 7; female, *n* = 30), the average baseline characteristics of the participants were: age, 35 ± 8 years (range 20–53); body weight, 84.9 ± 16.1 kg (range 59.9–114.3); and BMI, 30.5 ± 4.2 kg/m^2^ (range 24.2–40.3). The detailed characteristics are given in Table 1.

**Table 1:** Baseline characteristics of the study participants (n = 37)

Items	Values
Sex, *n* (%)	
Male	7 (19)
Female	30 (81)
Age, mean (SD), years	35 (8)
Highest education level achieved, *n* (%)	
High school graduate	14 (38)
College graduate	16 (43)
Post-graduate degree	7 (19)
Comorbidities, *n* (%)	
CVD history	1 (3)
Diabetes	7 (19)
Hypertension	3 (8)
Metabolic syndrome	10 (37)
Weight, mean (SD), kg	84.9 (16.1)
Body mass index, mean (SD), kg/m^2^	30.5 (4.2)

**Fig. 2:**
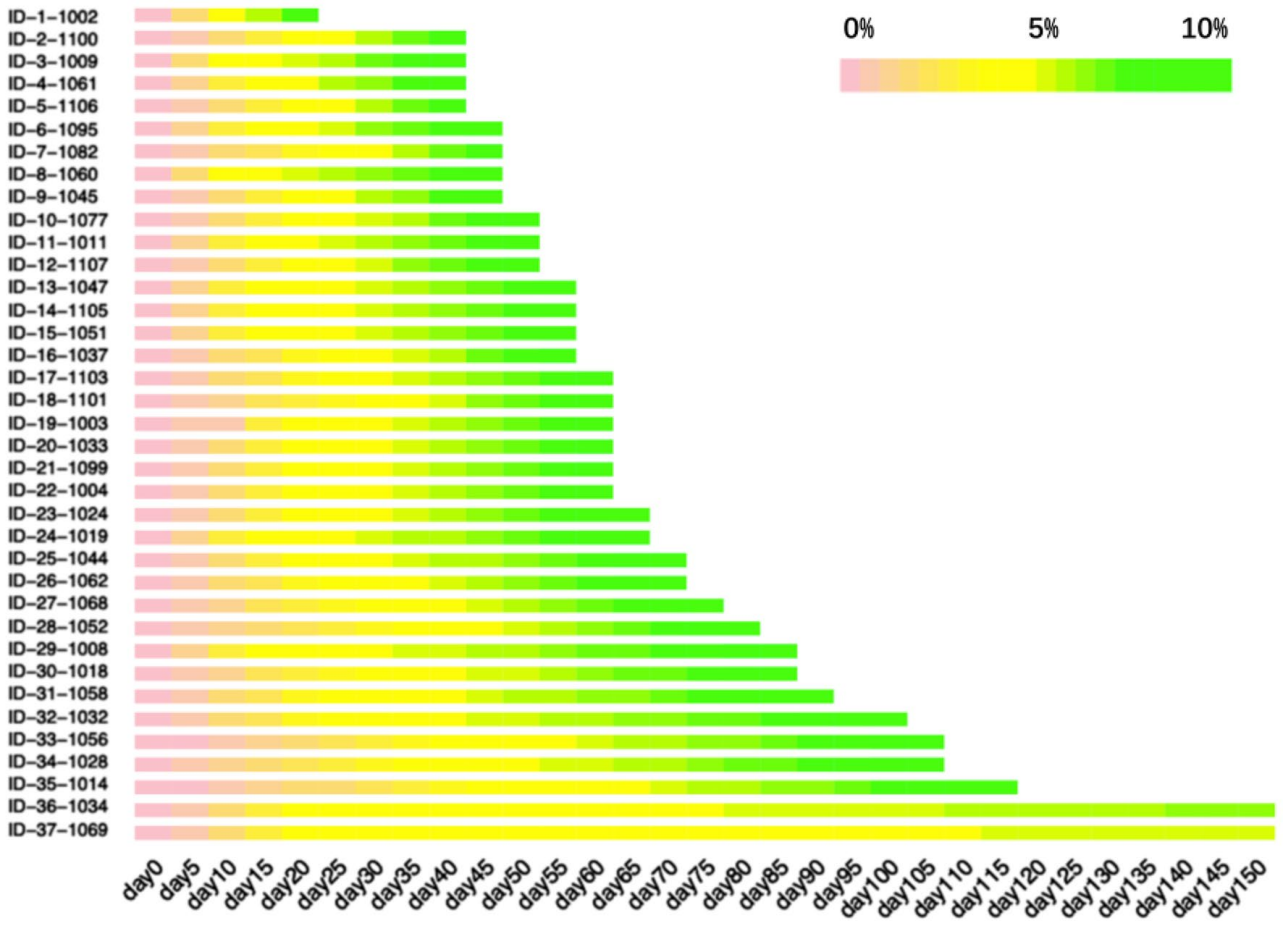
Velocity of body weight reduction over the course of the weight loss diet program

For the 71 participants who achieved a 5% reduction in body weight from baseline, the time interval ranged from 7 to 52 days, with a median of 20 days. For the 37 participants who attained a 10% reduction in body weight, the median time interval between the second and third visit was 39 days (ranging from 10 to 365 days). A heatmap plot which indicates the mean velocity of body weight reduction is shown in Fig. 2.

**Fig. 3:**
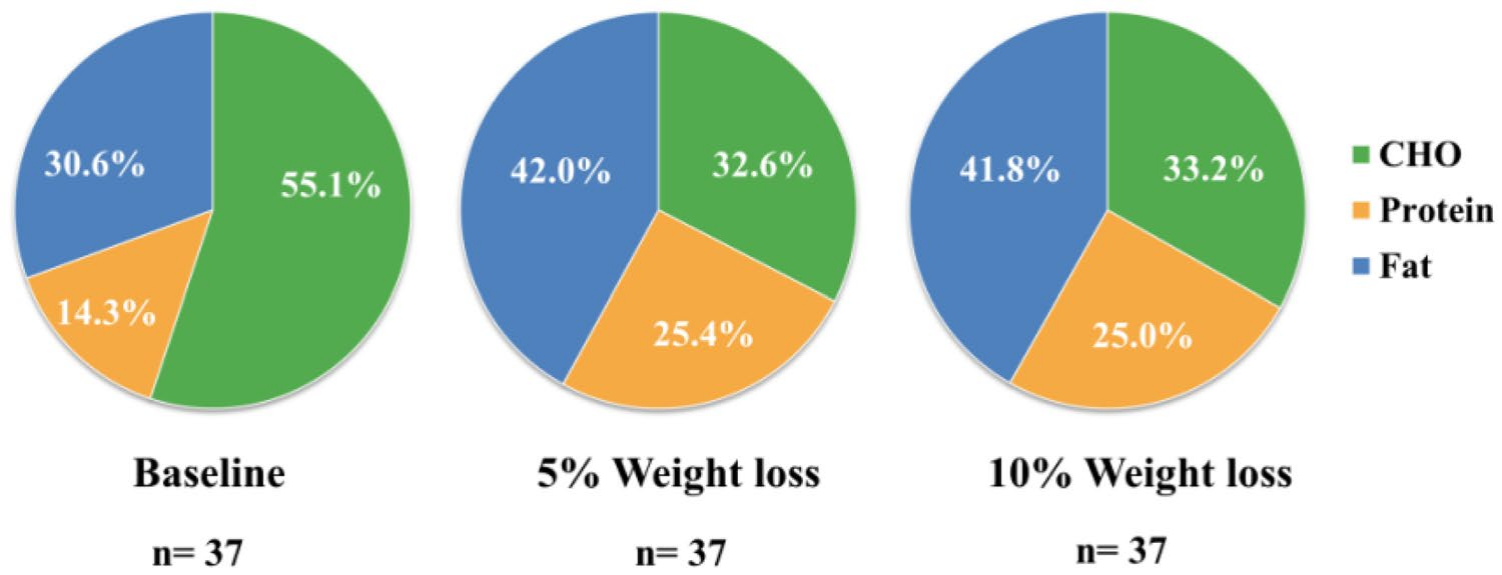
Macronutrient distribution at baseline, the 5% weight-loss visit, and 10% weight-loss visit

After following the intentional calorie-restricted (CR) diet plan for 2–3 months (average duration) and consequently achieving a successful 10% weight loss, the average daily calorie intakes of 37 subjects decreased from 1881.0 kcal to 1109.2 kcal, the percentage of their daily energy intake provided by protein increased from 14.3% to 25.0%, and the percentage of their daily energy provided by carbohydrate reduced from 55.1% to 33.2% (see Fig. 3). This indicated that the participants complied well with the dietitian-designed diet recommendations.

Regarding the nutrient intakes, we observed a substantial decline in the daily intake of carbohydrates (from 259.2 g/d to 98.2 g/d), together with a remarkable decrease in daily lipid intake (from 63.3 g/d to 50.0 g/d) (see Table 2). Participants were recommended to ensure adequate protein intakes (e.g., milk, beans, and their products); therefore, the calcium intake also increased remarkably, from 402.1 mg/d at baseline to 610.0 mg/d after 10% weight loss.

**Table 2:** Changes in dietary intake, body composition, inflammatory cytokines, and adipocytokines from baseline to 10% weight loss

	Baseline (*n* = 37)	10% weight loss (*n* = 37)	*p* value
Dietary intake			
Total energy intake (kcal/d)	1881.0 (650.2)	1109.2 (280.2)	0.000
Carbohydrates (g/d)	259.2 (106.0)	98.2 (45.0)	0.000
Protein (g/d)	66.7 (45.9, 81.8)	68.9 (56.7, 84.3)	0.492
Fat (g/d)	63.3 (48.1, 85.5)	50.0 (40.5, 61.1)	0.000
Calcium (mg/d)	402.1 (249.1, 545.2)	610.0 (483.5, 742.6)	0.024
Phosphorus (mg/d)	1019.8 (366.1)	965.8 (274.4)	0.398
Calcium/phosphorus ratio	0.45 (0.29, 0.65)	0.65 (0.52, 0.79)	0.009
Sunshine exposure (min/week)	60 (0, 210)	70 (0, 210)	0.670
Body composition			
Body weight (kg)	84.9 (16.1)	76.4(14.9)	0.000
BMI (kg/m^2^)	30.5 (4.2)	27.5 (3.9)	0.000
Fat body mass (kg)	30.3 (24.5, 37.3)	23.9 (19.8, 30.4)	0.000
Lean body mass (kg)	54.4 (9.9)	51.1 (8.9)	0.000
Body water (kg)	39.1 (7.2)	36.8 (6.4)	0.000
Minerals (kg)	4.7 (4.0, 5.5)	4.3 (3.6, 5.1)	0.000
Basal metabolism (kcal)	1300 (1236, 1471)	1272 (1211, 1420)	0.000
Serum markers			
IL-1β (pg/ mL)	5.91 (5.45, 7.08)	5.73 (5.27, 7.02)	0.886
IL-2 (pg/mL)	10.53 (8.94, 12.55)	9.37 (8.90, 10.66)	0.013
IL-6 (pg/ mL)	4.33 (2.05, 5.12)	3.89 (1.11, 5.41)	0.492
IL-8 (pg/ mL)	4.53 (3.82, 5.00)	4.58 (4.08, 5.01)	0.667
IL-10 (pg/mL)	8.82 (7.00, 12.20)	10.82 (7.49, 12.68)	0.274
TNF-α (pg/mL)	6.33 (5.34, 8.65)	6.72 (5.16, 7.49)	0.258
Leptin (ng/mL)	33.11 (18.58, 46.90)	18.9 (13.0, 30.8)	0.001
Adiponectin (μg/mL)	5.70 (4.60, 7.55)	6.08 (5.02, 8.15)	0.541
Acylated ghrelin (pg/mL)	11.46 (6.31, 19.57)	17.08 (6.50, 29.65)	0.033

Data with normal distributions are given as the mean (SD); those with skewed distributions are presented as the median (IQR)

As a result of closely adhering to the CR diet recipe, the 37 participants lost an average of 8.5 kg (10.0%) of weight compared with the baseline. Accompanied by weight reduction, their BMI, fat body mass, lean body mass, body water, minerals, and basal metabolism all decreased remarkably (see Table 2).

Regarding the inflammatory cytokines, a remarkable decrease in IL-2 (Δchange% 11.0%, *p* = 0.013) was observed after a 10% weight loss, whereas the changes in other cytokines (IL-1β, TNF-α, IL-6, IL-10, and IL-8) did not reach statistical significance. In contrast, remarkable changes in all adipocytokines were detected after the weight loss intervention, except for adiponectin. Serum leptin greatly reduced (Δchange% 42.9%, *p* = 0.001) after a 10% weight reduction. However, serum acylated ghrelin exhibited a remarkable increase (Δchange% − 49.0%, *p* = 0.033) at the 10% weight loss timepoint (Table 2).

After achieving the 10% weight loss, PINP, the marker of bone synthesis, decreased remarkably (Δchange% 17.2%, *p* = 0.000), whereas β-Crosslaps, the marker of bone absorption, did not exhibit remarkably change (see Table 3). Sexuality and hormone levels affect the bone turnover; therefore, we performed a subgroup analysis, and found that those two indicators remarkably changed in the female group when compared with the values at baseline. For female subjects, PINP decreased remarkably (Δchange% 17.6%, *p* = 0.000) and β-Crosslaps increased remarkably (Δchange% − 9.8%, *p* = 0.035), whereas for males, no remarkable changes were detected in either indicator.

**Table 3:** Changes in bone turnover indicators and bone metabolic parameters from baseline to 10% weight loss

	Baseline (*n* = 37)	10% weight loss (*n* = 37)	*p* value
PINP (ng/mL)			
Male	46.9 (32.9, 52.8)	45.0 (36.3, 49.9)	0.176
Female	46.5 (16.4)	38.3 (11.8)	0.000
Total	47.6 (18.7)	39.4 (12.0)	0.000
β-Crosslaps (ng/mL)			
Male	0.517 (0.165)	0.508 (0.174)	0.847
Female	0.285 (0.250, 0.330)	0.313 (0.296, 0.362)	0.035
Total	0.286 (0.252, 0.462)	0.327 (0.299, 0.429)	0.093
PTH (pg/mL)	88.7 (31.2)	73.3 (27.1)	0.001
25(OH) VitD (ng/mL)	16.9 (4.4)	20.0 (5.7)	0.001
Serum calcium (mmol/L)	2.32 (0.09)	2.38 (0.13)	0.053
Serum phosphorus (mmol/L)	1.17 (0.15)	1.23 (0.16)	0.025

Data with normal distributions are given as the mean (SD); those with skewed distributions are presented as the median (IQR)

The other two markers, PTH and 25(OH) VitD, which are closely associated with bone metabolism, also showed remarkable changes. We found a remarkable decrease (Δchange% 17.4%, *p* = 0.001) in serum PTH after 10% weight loss, in line with a remarkable increase (Δchange% − 18.3%, *p* = 0.001) in serum 25(OH) VitD. Subjects were stabilized at serum calcium levels of 2.32 mmol/L to 2.38 mmol/L. However, serum phosphorus levels increased slightly but remarkably, accompanied with weight loss (Δchange% − 5.1%, *p* = 0.025).

Table 4 summarizes the results of the Spearman bivariate correlations between changes in bone metabolic markers and the possible variables that might be explanatory. We found that ΔPINP was remarkably correlated to Δlean body mass (*r* = 0.418, *p* = 0.012) and to Δghrelin (*r* = − 0.374, *p* = 0.023). No remarkable correlations between Δβ-Crosslaps and other variables of interest were detectable. There were no remarkable correlations between ΔPTH and all other variables, except for ΔTNF-α (*r* = 0.366, *p* = 0.047). Δ25(OH) VitD was found to be positively correlated with ΔIL2 (*r* = 0.389, *p* = 0.027) and negatively correlated with Δghrelin (*r* = − 0.409, *p* = 0.017).

**Table 4:** Spearman correlations between changes in bone metabolic parameters and changes in possible influencing variables

	ΔPINP		Δβ-Crosslaps		ΔPTH		Δ25(OH) VitD	
	*r*	*p*	*r*	*p*	*r*	*p*	*r*	*p*
ΔFat body mass	− 0.004	0.982	0.256	0.181	− 0.176	0.345	− 0.022	0.905
ΔLean body mass	0.418 *	0.012	− 0.047	0.810	− 0.178	0.339	− 0.134	0.472
ΔCalcium intake	− 0.116	0.535	− 0.057	0.769	− 0.073	0.695	− 0.024	0.898
WL velocity	0.253	0.177	0.114	0.564	− 0.002	0.991	0.032	0.867
ΔIL1-β	− 0.081	0.670	0.002	0.991	0.242	0.198	− 0.232	0.218
ΔIL2	− 0.086	0.653	− 0.037	0.853	− 0.123	0.517	0.389 *	0.027
ΔIL6	0.178	0.347	− 0.053	0.788	0.238	0.205	0.039	0.836
ΔIL8	− 0.128	0.500	− 0.022	0.911	0.108	0.570	− 0.206	0.276
ΔIL10	− 0.083	0.663	− 0.016	0.935	0.143	0.449	− 0.063	0.743
ΔTNF-α	− 0.242	0.197	0.235	0.229	0.366*	0.047	0.038	0.840
ΔLeptin	0.181	0.338	0.067	0.735	0.087	0.649	− 0.077	0.687
ΔAdiponectin	− 0.015	0.936	− 0.136	0.491	− 0.145	0.445	− 0.202	0.285
ΔGhrelin	− 0.374*	0.023	0.018	0.927	0.13	0.495	− 0.409*	0.017

Δ variables were calculated via subtracting values at baseline from values assessed after 10% weight loss. Spearman correlation coefficients were computed to explore the correlations between 2 variables

*WL* weight loss

*p < 0.05

## Discussion

The current study was designed to determine whether bone turnover and bone metabolism-associated markers would change after a 10% weight loss and whether the changes in inflammatory cytokines and adipokines were correlated with the changes in bone metabolism markers. To the best of our knowledge, few investigations have been conducted to accurately measure the effects of a certain percentage of weight loss on bone metabolism; the relationships between bone metabolism markers and inflammatory, adipose-related cytokines have not been previously examined.

In the current study, our first key result was that the serum concentration of the bone resorption marker β-Crosslaps increased remarkably after 10% body weight was lost among female subjects. This finding is in agreement with previous studies [11–14] in which diet-induced weight loss also resulted in a rise in blood concentrations of β-Crosslaps in obese female subjects. A 2015 systematic review and meta-analysis [15] also reported the summarized data that 2- or 3-month interventions of weight loss triggered remarkable increases in the serum concentrations of β-Crosslaps (4.72 nmol/L; 95CI, 2.12–7.30 nmol/L). Some studies have reported that menstrual cycle might influence skeletal turnover. In this study, 12/30 women subjects said they had abnormal menstrual cycle at baseline. After achieving the 10% weight loss, 10 subjects told the researchers that their menstrual cycles had changed (becoming regular or irregular). We think the menstrual cycle change might be a potential factor mediating the effects of diet-induced weight loss on bone turnover. This speculation has been proved in a study which was performed to assess the effects of energy status and estrogen status on bone turnover [16]. The male subjects in this study did not exhibit remarkable changes in β-Crosslaps concentrations. This might be due to the insufficient sample of male participants, and is also likely because the androgens secreted by men help them to resist bone breakdown and maintain bone formation.

Another key finding of this study is that the participants, especially female subjects, exhibited remarkably decreased serum concentrations of PINP, a typical marker of bone formation, after their weights were reduced by 10%. This finding differed from the results of Kirsti Uusi-Rasi’s study [11], in which the mean increase (95% CI) in PINP was 28.2% (12.9–45.8%) in a high weight loss group, and 16.1% (2.2–31.8%) in a medium weight loss group after a 3 month intervention. Although these two contrasting results were both with female subjects, the age ranges were different, which might have resulted in differences in hormone levels. Together, the extent of energy restriction and detailed diet requirement of these two studies were not the same, which might have led to different levels of calcium intakes and different states of calcium and phosphorus sorption. PINP is a newly recognized indicator of bone formation; as such, there are very few studies which have used PINP as a main assessable outcome for comparisons.

In this study, weight loss was achieved through the prescription of a hypocaloric, diet. In response to this calorie-restricted diet, subjects reduced their body weight by 10% or more. Meanwhile, the diet pattern of subjects shifted to a high-protein, low-carbohydrate diet as dietitian required. Based on literature and nutritional theory, we think that compared to nutritional pattern, the intake of calcium and phosphorus, which are readily obtained from food, play a major role in bone remodeling and bone integrity maintaining [17, 18]. The results showed that calcium intake of 37 subjects increased significantly from 402.1 mg/d at baseline to 610.0 mg/d after 10% weight loss. One reason for this change might be that we list several high-protein foods such as milk and soybean products in the recommended diet items to ensure adequate protein intakes, at the same time these food groups are also rich in calcium. In addition, dietary phosphorus did not change significantly. We also investigated the sunshine exposure time of subjects and found that there was no significant difference before they started to lose weight and after 10% weight loss. We hypothesized the reason for the decreased bone formation and increased resorption occurred in the early stage of weight loss was energy restriction induced hormonal changes which might be expected to decrease bone mass. An additional rationale for this hypothesis is that energy restriction induced quick weight loss, which would lead to mechanical unloading of bone, with subsequently active bone turnover.

Notably, in our study, serum concentrations of PTH and 25(OH) VitD both exhibited remarkable changes favorable for bone health [PTH decreased, 25(OH)VitD increased] when weight was moderately reduced by a dietary intervention. In most previous weight loss studies [14, 19], bone loss was accompanied with an increase in serum PTH. This might be explained by the theory that a low dietary calcium intake, compounded by the stress of energy restriction and a reduced intake of other nutrients, causes a rise in PTH. Inconsistent with the results from the studies by Pamela S. Hinton [20] and Andrea R. Josse [21], serum PTH concentrations decreased during weight loss in our study, probably because the increased intake of calcium and more exposure to sunshine suppressed the increase in PTH. It is also possible that elevated concentrations of serum 25(OH) VitD in the course of weight loss given the mobilization of adipose stores [22] increased blood calcium and reducing PTH.

It is worth mentioning that a 10% weight reduction resulted in remarkably suppressed bone formation and increased bone breakdown in the context of elevated serum 25(OH)vitamin D concentrations and degraded serum PTH levels. This finding inspired us to pay more attention to the acute bone loss resulting from intentional weight reduction.

Obesity has been characterized as a state of chronic or low-grade systemic inflammation [23, 24]. Weight loss can not only improve hypertension, dyslipidemia, and insulin resistance, but can also lead to reductions in inflammatory biomarker concentrations [25–28] (CRP, TNF-a, IL-6, IL-8, etc.), and an increase in the expression of the anti-inflammatory markers adipokines [29, 30], especially in those investigations assessing a weight loss of at least 10%. Nevertheless, there were no remarkable changes found in most inflammatory biomarkers except for IL-2 in this study. This observation is probably due to the less sensitive detection method we used. According to the analysis by Ziqiang Li [31], the ELISA method this study employed is less sensitive than chemiluminescence immunoassays and flow cytometry immunofluorescence microsphere assays for the detection of inflammatory cytokines.

Another possible reason is that our data distribution was skewed; this is not favorable for discovering statistically remarkable differences between before and after weight loss treatment. Along with the decreases in total weight and body adiposity, the cytokines secreted by lipids, leptin, and ghrelin declined remarkably, and these findings were consistent with the results of previous studies [26, 32].

A reduction in adiposity during weight reduction diminishes circulating estrogen along with other sex hormones [33] and elevates sex-hormone-docking globulin. These changes may negatively influence bone osteoblast [34] and osteoblastic activity directly or indirectly [35] due to altered levels of cytokines (i.e., IL-2, IL-6, and tumor necrosis factor-α). In the current study, we found that changes in the bone formation marker PINP were positively correlated with changes in lean body mass and negatively correlated with changes in serum ghrelin. There has been no previous study examining the effect of serum cytokines on bone turnover markers; therefore, we could not compare the correlation analysis results with other research. However, several studies [36, 37] have reported that the changes in BMD in certain sites are linked to changes in lean body mass in adults. A similar association between changes in the bone formation marker PINP and changes in lean body mass was also found in our study. This sounds reasonable, because lean body mass consists of muscles and minerals; the latter is the basic bone structure material during the process of bone formation. Ghrelin has numerous physiological roles, for instance the induction of growth hormone release, as well as that of appetite, as well as fat aggregation [38]. One mechanism of diet-induced obesity is a rise in ghrelin resistance [39]. However, the inverse relationship between changes in PINP and changes in serum ghrelin found in this study needs further confirmation.

There are some setbacks to the present study. First, the sample size included in the analysis was small. Second, this study did not have an untreated control group. Finally, we did not measure sex hormones (such as estradiol, testosterone, etc.), which may influence bone metabolism to a large extent.

The first strength of the present research is its longitudinal design. Unlike other weight loss studies, which usually took the duration of weight loss treatment as the observation node, we used the percentage of weight loss as the follow-up node. Second, we paid attention to the early changes in bone metabolism during weight loss, providing a preventive perspective for bone health for weight loss individuals.

## Conclusions

In the present investigation, we established that bone formation was suppressed and bone absorption was increased in female subjects after a 10% weight loss. This suggests that women, both young or middle-aged, are at a greater risk of bone loss or fractures when losing weight. Notably, serum concentrations of PTH and 25(OH) VitD exhibited favorable changes when weight was moderately reduced by following a hypocaloric diet program but with high protein and adequate calcium. Bone formation was found to be associated with lean body mass and affected by ghrelin. Further investigations with larger sample sizes should be conducted to investigate the specific influencing factors of bone turnover in the process of diet-triggered weight loss to prevent the occurrence of early bone turnover slowdown.

## Supplementary Information

Below is the link to the electronic supplementary material.

Supplementary file1 (XLSX 36 KB)

## Data Availability

The data sets generated during and/or analyzed during the current study are available from the corresponding author on reasonable request.
